# Cardiac surgery receipt and outcomes for people using secondary mental healthcare services: Retrospective cohort study using a large mental healthcare database in South London

**DOI:** 10.1192/j.eurpsy.2022.2324

**Published:** 2022-10-04

**Authors:** Gonul Brooks, Ruwan Weerakkody, Matthew Harris, Robert Stewart, Gayan Perera

**Affiliations:** 1 Department of Psychological Medicine, Institute of Psychiatry, Psychology, and Neuroscience, King’s College London, London, United Kingdom; 2 Department of Vascular Surgery, The Royal Free Hospital, Pond Street, London NW3 2QG, United Kingdom; 3 NIHR Maudsley Biomedical Research Centre, South London and Maudsley NHS Foundation Trust, London, United Kingdom

**Keywords:** Cardiac surgery, Mental healthcare services, emergency admissions, length of stay

## Abstract

**Background:**

Patients diagnosed with mental health problems are more predisposed to cardiovascular disease, including cardiac surgery. Nevertheless, health outcomes after cardiac surgery for patients with mental health problems as a discrete group are unknown. This study examined the association between secondary care mental health service use and postoperative health outcomes following cardiac surgery.

**Methods:**

We conducted a retrospective observational research, utilizing data from a large South London mental healthcare supplier linked to national hospitalization data. OPCS-4 codes were applied to classify cardiac surgery. Health results were compared between those individuals with a mental health disorder diagnosis from secondary care and other local residents, including the length of hospital stay (LOS), inpatient mortality, and 30-day emergency hospital readmission.

**Results:**

Twelve thousand three hundred and eighty-four patients received cardiac surgery, including 1,481 with a mental disorder diagnosis. Patients with mental health diagnosis were at greater risk of emergency admissions for cardiac surgery (odds ratio [OR] 1.60; 1.43, 1.79), longer index LOS (incidence rate ratio 1.28; 1.26, 1.30), and at higher risk of 30-day emergency readmission (OR 1.53; 1.31, 1.78). Those who underwent pacemaker insertion and major open surgery had worse postoperative outcomes during index surgery hospital admission while those who had major endovascular surgery had worse health outcomes subsequent 30-day emergency hospital readmission.

**Conclusion:**

People with a mental health disorder diagnosis undertaking cardiac surgery have significantly worse health outcomes. Personalized guidelines and policies to manage preoperative risk factors require consideration and evaluation.

## Introduction

People with mental illness are approximately twice as likely to die from cardiovascular illness than the overall population [1]. A higher burden of modifiable cardiovascular risk factors (such as smoking and drug abuse), as well as inequalities in healthcare distribution, plays a potential role [2]. Patients diagnosed with mental health disorders, in general, are known to experience delayed presentation, diagnosis and treatment [3], poor communication with the healthcare system, and subsequently receive lower-quality care [4].

Of the 37,000 adults undergoing cardiac surgery annually in Britain, those suffering postoperative complications [5] have prolonged intensive care and hospitalization, higher 30-day readmission, and higher mortality [6, 7]. Approximately 20% of patients readmitted within 30 days after cardiac surgery [8] are at risk of higher rates of mortality, stroke, and cardiac transplantation than the general population [9].

Previous studies, including our own work, have suggested that postoperative surgical outcomes are commonly worse in patients with mental health problems [10, 11]. They may therefore constitute a potentially high-risk group for adverse outcomes after cardiac surgery. A better understanding of postoperative outcomes in this group would lead to more effective treatment and play a key role in managing cardiovascular disease [10], as well as contributing to understanding of broader health inequalities experienced.

In this study, we aimed to assess the risk of complications in a large sample of mental health service users undergoing cardiac surgery and in a comparison cohort from the same source population who had not used mental health services. Specifically, we sought to quantify cardiac operation standardized admission ratios (SARs) and to compare a broad selection of postoperative health outcomes between the cohorts. A secondary aim was to investigate these outcomes in four subgroups of cardiac surgery: major open cardiac surgery, major endovascular, cardiac supportive surgery, and pacemaker surgery.

## Methods

### Study setting and data source

We carried out a retrospective observational study, using data from the South London and Maudsley NHS Foundation Trust (SLaM) Biomedical Research Centre Case Register. SLaM provides mental healthcare to a catchment of approximately 1.36 million people across the London boroughs of Lambeth, Lewisham, Southwark, and Croydon [12]. The Clinical Record Interactive Search (CRIS) was created during 2007–2008 with National Institute for Health Research funding to allow the research use of deidentified data from SLaM’s electronic health record within a robust patient-led governance framework [13]. The register has subsequently been enhanced through natural language processing applications, obtaining structured data on a range of entities from text fields [13]. CRIS has also been linked to a number of different external data resources including national Hospital Episode Statistics (HES), which contains data on all admissions, Emergency Department attendances, and outpatients at NHS hospitals in England. Access to HES data was granted by NHS Digital for research purposes (https://digital.nhs.uk/). CRIS and its linked data have been approved for secondary analysis (Oxfordshire Research Ethics Committee C, reference 18/SC/0372), covering the data analysis described here.

### Sample

Utilizing the linkage between HES and CRIS, the sample comprised people who had a cardiac surgical procedure, were over the age of 18 at the time of this procedure, and were resident in the SLaM geographic catchment area during the years 2007–2019. Cardiac surgery operations were specified from HES using the OPCS-4 codes shown in Supplementary Table S1, and two cohorts were created: (a) Cohort 1: a comparison cohort of residents living in SLaM catchment boroughs who had no SLaM record since 2000 and had received cardiac surgery (*n* = 10,903); (b) Cohort 2: a case cohort of residents who had any mental health problem diagnosed at SLaM before receiving cardiac surgery (*n* = 1,481). We excluded patients who were in contact with SLaM without a mental health diagnosis (*n* = 32) and patients with absent data on age and gender (*n* = 18). When the cohorts were determined, types of cardiac surgery were ascertained and categorized into four subgroups: “cardiac supportive,” “major endovascular,” “major open,” and “pacemaker surgery.”

### Measurements

The index date was defined as the first date of entry to hospital for a cardiac operation between 1st January 2007 and 31st December 2019. Emergency hospital admissions were classified utilizing “admission methods” procedures 21–24 and 28, as per NHS data vocabulary definition [54]. Elective admissions were then identified from codes 11–13 [14]. We obtained demographic data on age, gender, ethnicity (categorized as White and non-White), and Index of Multiple Deprivation (IMD 2015) at the index date. IMD (2015) is a neighborhood-level statistic that measures income, occupation, healthcare and disabilities, education, skills, criminality, barriers to the accommodation, and services and living conditions [15]. IMD was applied to Lower Super Output Areas within the catchment area, a standard geographic unit covering 1,500–2,000 residents [15], based on the address at the time of the index date.

The following health outcomes were analyzed for the index admissions for cardiac surgery:

1. Type of hospital admission for cardiac surgical procedure (emergency or elective; odds ratio [OR]).
2. Length of hospital stay (LOS) from date of admission to date of discharge (incidence rate ratio [IRR] derived from Poisson regression models).
3. Inpatient mortality after cardiac operation, as recorded on HES (OR).

The following outcomes were ascertained following the index admission among individuals alive at discharge:

1. Readmission to hospital as an emergency within 30 days after leaving the hospital (OR).
2. LOS for emergency readmission as described in outcome four, from date of admission to date of discharge (IRR from zero-inflated Poisson regression models).

As mentioned, only patients with a mental health diagnosis were included in this study. The primary psychiatric condition at the time of initial mental health diagnosis was ascertained as follows: (a) dementia (F00, F01, F02, F03), (b) delirium (F05) or mild cognitive impairment (MCI, F06.7), (c) mental and behavioral disorders due to psychoactive substance use (F10–F19), (d) schizophrenia, schizotypal, delusional, and other non-mood psychotic disorders (F20–F29), (e) mood affective disorders (F30–F39), (f) anxiety, dissociate, stress-related, and somatoform and other nonpsychotic mental disorders (F40–F48), (g) behavioral syndromes associated with physiological disturbances and physical factors (F50–F59), and (h) disorders of adult personality and behavior (F60–F69).

### Statistical analysis

The cardiac surgery SARs adjusted for age and gender were ascertained for patients with mental health problems (cohort 2) who were admitted to hospital for cardiac surgery either via an elective or emergency route and compared against all adults who had received cardiac surgery (cohort 1 and cohort 2). When calculating SARs, all adults who had cardiac surgery, consisting of the SLaM catchment population (Lambeth, Southwark, Lewisham and Croydon residents), were used as the standard reference population. SAR for cardiac surgery was calculated between 2007 and 2019 covering an average of 98,106 annual adults (age 18 and over) residing in the SLaM catchment area (ONS population estimations). Out of this initial catchment population, 374,332 residents were in contact with SLaM service. Out of this sample of patients with mental health problems who used SLaM services and who underwent cardiac surgery (*n* = 1,481) were ascertained and included in SAR calculation in comparison to those all adults who had cardiac surgery in the SLaM catchment population. Indirect age- and gender-standardization methods were used to measure SARs by ascertaining age and gender on admission from the catchment and generating expected admission rates from the Census-derived age and gender structure of that source population.

Postoperative health outcomes were compared between the cohort with mental health problems (cohort 2) and the comparison cohort (cohort 1). Logistic regression analysis and subsequent OR calculations were conducted to examine associations with admissions to hospital via an emergency or elective admission, inpatient mortality, and whether patients were re-admitted to hospital via an emergency route within 30 days of discharge. IRRs from Poisson regression models measured associations with LOS for the index admission and for an emergency hospital readmission within 30 days. Unadjusted and adjusted associations which were adjusted for age and gender, ethnicity and IMD score were calculated. This study was designed as a descriptive, rather than causal or predictive study; therefore, associations were adjusted simply for demographic factors rather than for wider ranges of potential confounders [16]. Statistical analyses were conducted using STATA 13 software.

## Results

Table 1 presents the demographic and clinical characteristics for both cohorts: 1,481 people diagnosed with mental health disorders who had cardiac surgery (cohort 2) and the comparison cohort of 10,903 catchment residents without mental health problems, who had cardiac surgery (cohort 1). The most frequent method of admission for cardiac surgery was elective, which was more common in the comparison cohort (67.8%) than the case cohort (57.4%). Patients with mental health problems were slightly older and more likely to be female and from a White ethnic group but did not differ significantly from the comparison cohort in neighborhood IMD score. The most common diagnostic group in patients with mental health problems was mood disorders (24.5%). Cardiac surgery SARs for the cohort of patients with mental health problems were 1.28 (95% CI: 1.22, 1.35) overall, and 1.57 (95% CI: 1.45, 1.69) and 1.13 (95% CI; 1.05, 1.20) for emergency and elective surgery, respectively.

**Table 1.** Characteristics of the cohorts.

Characteristics	Comparison cohort (cohort 1) (*n* = 10,903)	Mental health cohort (cohort 2) (*n* = 1,481)	*p*-value for difference
*Method of admission for cardiac surgery* (%)			<0.001
Elective	7,390 (67.8)	850 (57.4)	
Emergency	3,513 (32.2)	631 (42.6)	
*Mean age (SD)*	65.9 (19.7)	68.0 (17.5)	<0.001
*Gender* (%)			<0.001
Female	3,765 (34.5)	576 (38.9)	
Male	7,138 (65.5)	905 (61.1)	
*Ethnicity* (%)			<0.001
White	6,519 (59.8)	1,064 (71.8)	
Non-White	4,384 (40.2)	417 (28.2)	
*Mean IMD 2015 score (SD)*	27.3 (11.1)	27.2 (11.7)	0.75
*Initial primary mental health diagnosis* (%)			
Dementia (F00–F03)		290 (19.6)	
Delirium or MCI (F05 or F06.7)		195 (13.2)	
Mental and behavioral disorders due to psychoactive substance use (F10–F19)		200 (13.5)	
Schizophrenia, schizotypal, delusional, and other non-mood psychotic disorders (F20–F29)		92 (6.2)	
Mood [affective] disorders (F30–F39)		363 (24.5)	
Anxiety, dissociative, stress-related, somatoform, and other nonpsychotic mental disorders (F40–F48)		227 (15.3)	
Behavioral syndromes associated with physiological disturbances and physical factors (F50–F59)		34 (2.3)	
Disorders of adult personality and behavior (F60–F69)		26 (1.8)	
*Type of cardiac surgery* (%)			<0.001
Cardiac supportive surgery	450 (4.1)	74 (5.0)	
Major endovascular	106 (1.0)	22 (1.5)	
Major open	5,237 (48.0)	416 (28.1)	
Pacemaker surgery	5,110 (46.9)	969 (65.4)	
*Outcomes during index hospitalization*			
Mean duration of hospitalization in days (SD)	10.5 (28.9)	13.1 (26.1)	<0.001
Inpatient mortality (%)	454 (4.2)	68 (4.6)	0.47
*Outcomes within 30-days discharge from cardiac surgery*			
Emergency hospital readmission (%)	1,171 (10.7)	229 (15.5)	<0.001
Mean duration in days of emergency hospital readmission (SD)	7.8 (21.9)	7.2 (10.7)	0.29

*Note:* Patients from general population who had cardiac surgery in the catchment population without any contact with SLaM.

Abbreviation: SLaM, South London and Maudsley NHS Foundation Trust.

Table 2 displays further analyses of postoperative health outcomes. After adjusting for sociodemographic factors, those with mental health problems were more likely to have an emergency admission and had a longer index LOS but did not differ significantly on inpatient mortality; they were more likely to be readmitted to hospital via an emergency route within 30 days but did not have a significantly longer LOS on their readmission.

**Table 2.** Post-operative outcomes for cardiac surgical patients with MHP compared with comparison population* [Ratio (95% confidence Interval)].

	Index cardiac surgery hospitalization			Subsequent readmission	
Adjustments	Odds ratio for emergency route of admission	Incidence rate ratio for length of stay	Odds ratio for mortality during index admission	Odds ratio for readmission within 30 days	Incidence rate ratio for length of stay of 30-day readmission
Univariate (unadjusted)	1.56 (1.40, 1.74)	1.25 (1.23, 1.27)	1.11 (0.85, 1.44)	1.52 (1.30, 1.71)	1.00 (0.95, 1.05)
Adjusted (*n* = 12,384)^a^	1.60 (1.43, 1.79)	1.28 (1.26, 1.30)	1.15 (0.88, 1.49)	1.53 (1.31, 1.78)	0.99 (0.94, 1.04)

*Note:* Comparison cohort include patients from general population who had cardiac surgery in the catchment population without any contact with SLaM.

a Adjusted for age, gender, ethnicity, and IMD score.

Abbreviation: SLaM, South London and Maudsley NHS Foundation Trust.

Table 3 summarizes the postoperative cardiac surgical results classified by the four types of cardiac surgery. In the fully adjusted models, those with mental health problems in all four subtypes of cardiac surgery showed significantly higher odds of 30-day hospital emergency readmissions, but no significant associations were found in inpatient mortality outcome. Health outcomes following major endovascular surgery were not significantly different between cohorts in terms of index cardiac surgery hospitalization outcomes. However, those with mental health problems had significantly longer length of stay subsequent to index hospitalization. Following major open surgery and pacemaker surgery, patients with mental health problems had significantly higher odds of emergency admission and longer length of stay for the index procedure.

**Table 3.** Post-operative cardiac surgery outcomes for patients MHP with compared with those from the general population by type of cardiac surgery OR/ IRR (95% CI), *p*-value^a^.

	Index cardiac surgery hospitalization			Subsequent readmission	
Type of cardiac surgery	Odds ratio for emergency route of admission	Incidence rate ratio for length of stay	Odds ratio for mortality during index admission	Odds ratio for readmission within 30 days	Incidence rate ratio for length of stay of 30-day readmission
Cardiac supportive surgery (*n* = 524)	1.09 (0.63, 1.88)	1.97 (1.87, 2.08)	1.07 (0.63, 1.81)	2.51 (1.21, 5.18)	0.66 (0.45, 0.98)
Major endovascular surgery (*n* = 128)	1.11 (0.42, 2.92)	1.02 (0.89, 1.18)	0.60 (0.06, 5.77)	3.64 (1.05, 12.67)	2.54 (1.24, 5.21)
Major open (*n* = 5,653)	1.72 (1.13, 1.99)	1.23 (1.20, 1.26)	1.46 (0.90, 2.39)	1.45 (1.24, 2.09)	0.95 (0.87, 1.05)
Pacemaker surgery (*n* = 6,079)	1.65 (1.22, 1.82)	1.56 (1.53, 1.60)	1.13 (0.70, 1.83)	1.51 (1.31, 1.98)	0.98 (0.91, 1.05)

a Adjusted for age, gender, ethnicity, IMD score.

Abbreviation: IMD, index of multiple deprivation.

## Discussion

This study examined admission rates and postoperative health outcomes for cardiac surgery in people with mental health problems compared to residents from the same catchment population receiving these procedures. The primary investigation found a significantly raised SAR for both emergency and elective cardiac surgery admissions associated with mental health problems. Furthermore, patients with mental health problems were more likely to experience the cardiac surgery in the context of an emergency admission and also had worse postoperative outcomes including longer LOS for the index admission and a greater risk of 30-day emergency hospital readmissions. Previous studies have shown that people with mental health problems increased emergency hospitalization [17–19] and longer LOS following cardiac surgery [20], and they are also more likely to increase emergency hospitalization [17–19] and increased risk of 30-day readmission [20–22].

Considering the index hospitalization, patients with mental health problems were substantially more likely to be admitted via an emergency route and remain in hospital longer. Both these findings are consistent with what is known of disparities in healthcare access and a tendency to present with more advanced disease [23]. With regard to the observed longer hospitalization, causal pathways cannot be discerned from the available data; however, it may be partly a function of a higher rate of emergency presentation, which would be associated with a higher perioperative risk, as well as mental health and complex social factors that may need resolution prior to discharge from hospital. Mortality during the index hospitalization did not differ significantly between the cohorts; however, data were not available on longer-term mortality in the comparison group to allow full evaluation.

We subclassified four broad groups of cardiac procedures: cardiac supportive, major endovascular, major open, and pacemaker (with major open surgery and pacemaker procedures comprising the majority). In each of these subgroups, except for major endovascular procedures, postoperative health outcomes showed inequalities in patients with mental health problems, with longer LOS, and increased risk of readmission within 30 days. Endovascular surgery is less invasive than major open surgery [24], inherently posing a lower physiological risk; thus, it is conceivable that differences in outcome in this group may not manifest to the same extent. In the group receiving a pacemaker, although also a less-invasive procedure, mental health problems were associated with a significantly higher likelihood of emergency admission, which is a risk factor for increased length of stay and postoperative complications.

We observed the mean age of both cohorts to be 65 and over. Emergency admissions for cardiac surgery have previously been found to be associated with worse postoperative outcomes among elderly patients with mental health problems, thought to be because of delayed diagnosis and referral by primary care service providers [25]. Several potential characteristics may underlie worse outcomes among people mental health problems, including difficulty expressing symptoms, poor self-monitoring [26, 27], age [28], and lack of recognition of illness [29]. Other factors may include difficulties interacting with clinicians concerning symptoms, higher emergency surgery rates, struggling with follow-up, poor accessibility [30–32], and delays in diagnosis of post-surgery complications.

The main strength of this study was the deployment of naturalistic, large-scale data, based on an ethnically diverse, varied population with long follow-up data. It is worth noting some limitations. This study pertains to a single geographical area and therefore may to an extent reflect local service provision within that catchment. Moreover, SLaM will not have complete ascertainment of all mental health disorders for its source populations (many patients with minor or short-lived mental health conditions will have been managed by their own GP without specialist care contact). Additionally, as with any large-scale health datasets, there will be inevitable coding errors (though this would be mitigated by the large sample size), and lack of detail with regard to specific patient-level factors precluded the investigation of processes underlying observed differences between the cohorts [33].

Future research might helpfully seek to extract more detailed information on perioperative care for these procedures to verify why increased risks might have been observed in those with mental health problems. Proactive interventions for people with mental health problems and other vulnerable groups may support surgical recovery. In addition, targeted preoperative assessment and prevention may be indicated to diminish the risk of adverse outcomes following surgery [21] and help address inequalities. Finally, increased input from mental health professionals could regularly be included in integrated surgical healthcare teams across the healthcare system, to provide patients with proactive interventions following surgery.

## Supplementary material

For supplementary material accompanying this paper visit https://doi.org/10.1192/j.eurpsy.2022.2324. click here to view supplementary material
