# Supplementary material for: Cardiac surgery receipt and outcomes for people using secondary mental healthcare services: Retrospective cohort study using a large mental healthcare database in South London
Source: Eur Psychiatry. 2022 Oct 4;65(1):e67. doi: 10.1192/j.eurpsy.2022.2324 (PMC9677442; doi:10.1192/j.eurpsy.2022.2324)
Supplement: Supplementary file 1 [file S0924933822023240sup001.docx]

**Supplementary Table 1**

| **OPCS procedure code for Cardiac surgery** | **OPCS procedure description** | **Cardiac surgery group** |
| --- | --- | --- |
| K023 | Implantation of prosthetic heart | Cardiac supportive surgery |
| K541 | Open implantation of ventricular assist device | Cardiac supportive surgery |
| K548 | Other specified open heart assist operations | Cardiac supportive surgery |
| K549 | Unspecified open heart assist operations | Cardiac supportive surgery |
| K569 | TRANSLUMINAL HEART ASSIST OPERATIONS (U) | Cardiac supportive surgery |
| K562 | Transluminal insertion of heart assist system NEC | Cardiac supportive surgery |
| Y494 | Transapical approach to heart | Major endovascular |
| Y794 | Transluminal approach to organ through aortic artery | Major endovascular |
| Y792 | Transluminal approach to organ through brachial artery | Major endovascular |
| Y793 | Transluminal approach to organ through femoral artery | Major endovascular |
| Y791 | Transluminal approach to organ through subclavian artery | Major endovascular |
| Y798 | Other specified approach to organ through artery | Major endovascular |
| K335 | Aortic root replacement NEC | Major open |
| K333 | Aortic root replacement using homograft | Major open |
| K334 | Aortic root replacement using mechanical prosthesis | Major open |
| K331 | Aortic root replacement using pulmonary valve autograft with right ventricle to pulmonary artery valved conduit | Major open |
| K338 | Other specified operations on aortic root | Major open |
| K339 | Unspecified operations on aortic root | Major open |
| L212 | Bypass of segment of thoracic aorta by anastomosis of aorta to aorta NEC | Major open |
| L202 | Emergency bypass of segment of thoracic aorta by anastomosis of aorta to aorta NEC | Major open |
| L182 | Emergency replacement of aneurysmal segment of thoracic aorta by anastomosis of aorta to aorta NEC | Major open |
| L254 | Operations on aneurysm of aorta NEC | Major open |
| L238 | Other specified plastic repair of aorta | Major open |
| L231 | Plastic repair of aorta and end to end anastomosis of aorta | Major open |
| L236 | Plastic repair of aorta and insertion of tube graft | Major open |
| L224 | Removal of prosthesis from aorta | Major open |
| L192 | Replacement of aneurysmal segment of thoracic aorta by anastomosis of aorta to aorta NEC | Major open |
| L235 | Revision of plastic repair of aorta | Major open |
| L221 | Revision of prosthesis of thoracic aorta | Major open |
| L239 | Unspecified plastic repair of aorta | Major open |
| L228 | ATTENTION TO PROSTHESIS OF AORTA (O) | Major open |
| L229 | ATTENTION TO PROSTHESIS OF AORTA (U) | Major open |
| L251 | ENDARTERECTOMY OF AORTA AND PATCH REPAIR OF AORTA | Major open |
| L252 | ENDARTERECTOMY OF AORTA NEC | Major open |
| L255 | OPERATIONS ON AORTIC BODY | Major open |
| L258 | OTHER OPEN OPERATIONS ON AORTA (O) | Major open |
| L259 | OTHER OPEN OPERATIONS ON AORTA (U) | Major open |
| L198 | OTHER REPLACEMENT OF ANEURYSMAL SEGMENT OF AORTA (O) | Major open |
| L199 | OTHER REPLACEMENT OF ANEURYSMAL SEGMENT OF AORTA (U) | Major open |
| L208 | Other specified other emergency bypass of segment of aorta | Major open |
| L188 | Repair of leaking aneurysm of arch of aorta | Major open |
| L189 | Unspecified emergency replacement of aneurysmal segment of aorta | Major open |
| L209 | Unspecified other emergency bypass of segment of aorta | Major open |
| L216 | BYPASS OF BIFURCATION OF AORTA BY ANASTOMOSIS OF AORTA TO IL | Major open |
| L215 | BYPASS OF SEGMENT OF ABDOMINAL AORTA BY ANASTOMOSIS OF AORTA | Major open |
| L214 | BYPASS OF SEGMENT OF INFRARENAL ABDOMINAL AORTA BY ANASTOMOS | Major open |
| L213 | BYPASS OF SEGMENT OF SUPRARENAL ABDOMINAL AORTA BY ANASTOMOS | Major open |
| L206 | Emergency bypass of bifurcation of aorta by anastomosis of aorta to iliac artery NEC | Major open |
| L205 | Emergency bypass of segment of abdominal aorta by anastomosis of aorta to aorta NEC | Major open |
| L204 | Emergency bypass of segment of infrarenal abdominal aorta by anastomosis of aorta to aorta NEC | Major open |
| L203 | Emergency bypass of segment of suprarenal abdominal aorta by anastomosis of aorta to aorta NEC | Major open |
| L186 | Emergency replacement of aneurysmal bifurcation of aorta by anastomosis of aorta to iliac artery | Major open |
| L185 | Emergency replacement of aneurysmal segment of abdominal aorta by anastomosis of aorta to aorta NEC | Major open |
| L184 | Emergency replacement of aneurysmal segment of infrarenal abdominal aorta by anastomosis of aorta to aorta | Major open |
| L183 | Emergency replacement of aneurysmal segment of suprarenal abdominal aorta by anastomosis of aorta to aorta | Major open |
| L253 | OPEN EMBOLECTOMY OF BIFURCATION OF AORTA | Major open |
| L218 | OTHER BYPASS OF SEGMENT OF AORTA (O) | Major open |
| L219 | OTHER BYPASS OF SEGMENT OF AORTA (U) | Major open |
| L196 | REPLACEMENT OF ANEURYSMAL BIFURCATION OF AORTA BY ANASTOMOSIS | Major open |
| L195 | REPLACEMENT OF ANEURYSMAL SEGMENT OF ABDOMINAL AORTA BY ANASTOMOSIS | Major open |
| L194 | REPLACEMENT OF ANEURYSMAL SEGMENT OF INFRARENAL ABDOMINAL AORTA | Major open |
| L193 | REPLACEMENT OF ANEURYSMAL SEGMENT OF SUPRARENAL ABDOMINAL AORTA | Major open |
| L223 | REVISION OF PROSTHESIS OF ABDOMINAL AORTA NEC | Major open |
| L222 | REVISION OF PROSTHESIS OF BIFURCATION OF AORTA | Major open |
| L211 | Bypass of segment of ascending aorta by anastomosis of aorta to aorta NEC | Major open |
| K385 | Closure of aortic sinus of valsalva fistula | Major open |
| L201 | Emergency bypass of segment of ascending aorta by anastomosis of aorta to aorta NEC | Major open |
| L181 | Emergency replacement of aneurysmal segment of ascending aorta by anastomosis of aorta to aorta | Major open |
| K551 | Ligation of sinus of valsalva | Major open |
| K386 | Repair of aortic sinus of valsalva aneurysm | Major open |
| L191 | Replacement of aneurysmal segment of ascending aorta by anastomosis of aorta to aorta NEC | Major open |
| K523 | Endocardial excision of rhythmogenic focus | Major open |
| K522 | Epicardial excision of rhythmogenic focus | Major open |
| K526 | Incision of tissue in atria | Major open |
| K521 | Open ablation of atrioventricular node | Major open |
| K524 | Open division of accessory pathway within heart | Major open |
| K525 | Open division of conducting system of heart NEC | Major open |
| K528 | Other specified open operations on conducting system of heart | Major open |
| K228 | Other specified other operations on wall of atrium | Major open |
| K529 | OPEN OPERATIONS ON CONDUCTING SYSTEM OF HEART (U) | Major open |
| K223 | Exclusion of left atrial appendage NEC | Major open |
| K108 | Other specified repair of defect of interatrial septum | Major open |
| K104 | Primary repair of defect of interatrial septum NEC | Major open |
| K102 | Repair of defect of interatrial septum using pericardial patch | Major open |
| K101 | Repair of defect of interatrial septum using prosthetic patch | Major open |
| K103 | Repair of defect of interatrial septum using tissue graft NEC | Major open |
| K105 | Revision of repair of defect of interatrial septum | Major open |
| K109 | Unspecified repair of defect of interatrial septum | Major open |
| K424 | Allograft replacement of four or more coronary arteries | Major open |
| K421 | Allograft replacement of one coronary artery | Major open |
| K423 | Allograft replacement of three coronary arteries | Major open |
| K422 | Allograft replacement of two coronary arteries | Major open |
| K454 | Anastomosis of mammary artery to coronary artery NEC | Major open |
| K453 | Anastomosis of mammary artery to left anterior descending coronary artery | Major open |
| K455 | Anastomosis of thoracic artery to coronary artery NEC | Major open |
| K414 | Autograft replacement of four or more coronary arteries NEC | Major open |
| K411 | Autograft replacement of one coronary artery NEC | Major open |
| K413 | Autograft replacement of three coronary arteries NEC | Major open |
| K412 | Autograft replacement of two coronary arteries NEC | Major open |
| K451 | Double anastomosis of mammary arteries to coronary arteries | Major open |
| K452 | Double anastomosis of thoracic arteries to coronary arteries NEC | Major open |
| K461 | Double implantation of mammary arteries into heart | Major open |
| K462 | Double implantation of thoracic arteries into heart NEC | Major open |
| K471 | Endarterectomy of coronary artery | Major open |
| K463 | Implantation of mammary artery into heart NEC | Major open |
| K464 | Implantation of thoracic artery into heart NEC | Major open |
| K428 | Other specified allograft replacement of coronary artery | Major open |
| K458 | Other specified connection of thoracic artery to coronary artery | Major open |
| K418 | Other specified other autograft replacement of coronary artery | Major open |
| K468 | Other specified other bypass of coronary artery | Major open |
| K448 | Other specified other replacement of coronary artery | Major open |
| K438 | Other specified prosthetic replacement of coronary artery | Major open |
| K478 | Other specified repair of coronary artery | Major open |
| K408 | Other specified saphenous vein graft replacement of coronary artery | Major open |
| K434 | Prosthetic replacement of four or more coronary arteries | Major open |
| K431 | Prosthetic replacement of one coronary artery | Major open |
| K433 | Prosthetic replacement of three coronary arteries | Major open |
| K432 | Prosthetic replacement of two coronary arteries | Major open |
| K473 | Repair of aneurysm of coronary artery | Major open |
| K472 | Repair of arteriovenous fistula of coronary artery | Major open |
| K475 | Repair of arteriovenous malformation of coronary artery | Major open |
| K474 | Repair of rupture of coronary artery | Major open |
| K441 | Replacement of coronary arteries using multiple methods | Major open |
| K456 | Revision of connection of thoracic artery to coronary artery | Major open |
| K465 | Revision of implantation of thoracic artery into heart | Major open |
| K442 | Revision of replacement of coronary artery | Major open |
| K404 | Saphenous vein graft replacement of four or more coronary arteries | Major open |
| K401 | Saphenous vein graft replacement of one coronary artery | Major open |
| K403 | Saphenous vein graft replacement of three coronary arteries | Major open |
| K402 | Saphenous vein graft replacement of two coronary arteries | Major open |
| K429 | Unspecified allograft replacement of coronary artery | Major open |
| K459 | Unspecified connection of thoracic artery to coronary artery | Major open |
| K419 | Unspecified other autograft replacement of coronary artery | Major open |
| K469 | Unspecified other bypass of coronary artery | Major open |
| K449 | Unspecified other replacement of coronary artery | Major open |
| K439 | Unspecified prosthetic replacement of coronary artery | Major open |
| K479 | Unspecified repair of coronary artery | Major open |
| K409 | Unspecified saphenous vein graft replacement of coronary artery | Major open |
| K443 | Other replacement of coronary artery | Major open |
| K444 | Other replacement of coronary artery | Major open |
| X581 | Extracorporeal membrane oxygenation | Cardiac supportive surgery |
| K561 | Transluminal insertion of pulsation balloon into aorta | Cardiac supportive surgery |
| K248 | Other specified other operations on ventricles of heart | Major open |
| K235 | Partial left ventriculectomy | Major open |
| K244 | Repair of left ventricular aneurysm | Major open |
| K243 | Repair of right ventricular aneurysm | Major open |
| K234 | Revascularisation of wall of heart | Major open |
| K246 | Myectomy of left ventricular outflow tract | Major open |
| K247 | Myotomy of left ventricular outflow tract | Major open |
| K248 | Other specified other operations on ventricles of heart | Major open |
| K245 | Relief of left ventricular outflow tract obstruction | Major open |
| K244 | Repair of left ventricular aneurysm | Major open |
| K243 | Repair of right ventricular aneurysm | Major open |
| K373 | Repair of subaortic stenosis | Major open |
| K232 | Biopsy of lesion of wall of heart | Major open |
| K236 | Cardiomyoplasty | Major open |
| K221 | Excision of lesion of atrium | Major open |
| K671 | Excision of lesion of pericardium | Major open |
| K231 | Excision of lesion of wall of heart NEC | Major open |
| K484 | Exploration of coronary artery | Major open |
| K532 | Exploration of heart NEC | Major open |
| T031 | Exploratory median sternotomy | Major open |
| K692 | Fenestration of pericardium | Major open |
| K691 | Freeing of adhesions of pericardium | Major open |
| K531 | Inspection of valve of heart | Major open |
| K483 | Open angioplasty of coronary artery | Major open |
| K553 | Open removal of cardiac thrombus | Major open |
| K554 | Open removal of cardiac vegetations NEC | Major open |
| K678 | Other specified excision of pericardium | Major open |
| K698 | Other specified incision of pericardium | Major open |
| L808 | Other specified operations on individual pulmonary veins | Major open |
| K538 | Other specified other incision of heart | Major open |
| K488 | Other specified other open operations on coronary artery | Major open |
| K558 | Other specified other open operations on heart | Major open |
| K238 | Other specified other operations of wall of heart | Major open |
| K388 | Other specified other operations on structure adjacent to valve of heart | Major open |
| K128 | Other specified repair of defect of unspecified septum of heart | Major open |
| K124 | Primary repair of defect of septum of heart NEC | Major open |
| K222 | Repair of atrium NEC | Major open |
| K122 | Repair of defect of septum of heart using pericardial patch NEC | Major open |
| K121 | Repair of defect of septum of heart using prosthetic patch NEC | Major open |
| K123 | Repair of defect of septum of heart using tissue graft NEC | Major open |
| L801 | Repair of pulmonary vein stenosis | Major open |
| K556 | Repair of traumatic injury of heart | Major open |
| K233 | Repair of wall of heart NEC | Major open |
| K555 | Resection of heart tumour | Major open |
| K125 | Revision of repair of septum of heart NEC | Major open |
| K481 | Transection of muscle bridge of coronary artery | Major open |
| K482 | Transposition of coronary artery NEC | Major open |
| K679 | Unspecified excision of pericardium | Major open |
| K699 | Unspecified incision of pericardium | Major open |
| K539 | Unspecified other incision of heart | Major open |
| K129 | Unspecified repair of defect of unspecified septum of heart | Major open |
| K489 | Other open op coronary art NOS | Major open |
| K239 | OTHER OPERATIONS OF WALL OF HEART (U) | Major open |
| K389 | OTHER OPERATIONS ON STRUCTURE ADJACENT TO VALVE OF HEART (U) | Major open |
| L809 | Unspecified operations on individual pulmonary veins | Major open |
| K249 | Unspecified other operations on ventricles of heart | Major open |
| K229 | Unspecified other operations on wall of atrium | Major open |
| K559 | Unspecified other open operations on heart | Major open |
| K617 | Implantation of biventricular cardiac pacemaker system | Pacemaker surgery |
| K611 | Implantation of cardiac pacemaker system NEC | Pacemaker surgery |
| K616 | Implantation of dual chamber cardiac pacemaker system | Pacemaker surgery |
| K607 | Implantation of intravenous biventricular cardiac pacemaker system | Pacemaker surgery |
| K601 | Implantation of intravenous cardiac pacemaker system NEC | Pacemaker surgery |
| K606 | Implantation of intravenous dual chamber cardiac pacemaker system | Pacemaker surgery |
| K605 | Implantation of intravenous single chamber cardiac pacemaker system | Pacemaker surgery |
| K615 | Implantation of single chamber cardiac pacemaker system | Pacemaker surgery |
| K618 | Other specified other cardiac pacemaker system | Pacemaker surgery |
| K619 | Unspecified other cardiac pacemaker system | Pacemaker surgery |
| K591 | Implantation of cardioverter defibrillator using one electrode lead | Pacemaker surgery |
| K596 | Implantation of cardioverter defibrillator using three electrode leads | Pacemaker surgery |
| K592 | Implantation of cardioverter defibrillator using two electrode leads | Pacemaker surgery |
| K608 | Other specified cardiac pacemaker system introduced through vein | Pacemaker surgery |
| K598 | Other specified cardioverter defibrillator introduced through the vein | Pacemaker surgery |
| K614 | Removal of cardiac pacemaker system NEC | Pacemaker surgery |
| K595 | Removal of cardioverter defibrillator | Pacemaker surgery |
| K604 | Removal of intravenous cardiac pacemaker system | Pacemaker surgery |
| K613 | Renewal of cardiac pacemaker system NEC | Pacemaker surgery |
| K594 | Renewal of cardioverter defibrillator | Pacemaker surgery |
| K603 | Renewal of intravenous cardiac pacemaker system | Pacemaker surgery |
| K612 | Resiting of lead of cardiac pacemaker system NEC | Pacemaker surgery |
| K593 | Resiting of lead of cardioverter defibrillator | Pacemaker surgery |
| K602 | Resiting of lead of intravenous cardiac pacemaker system | Pacemaker surgery |
| K609 | Unspecified cardiac pacemaker system introduced through vein | Pacemaker surgery |
| K599 | Unspecified cardioverter defibrillator introduced through the vein | Pacemaker surgery |
| K724 | Removal of subcutaneous cardioverter defibrillator | Pacemaker surgery |
| K743 | Renewal of biventricular cardiac pacemaker NEC | Pacemaker surgery |
| K742 | Renewal of dual chamber cardiac pacemaker system NEC | Pacemaker surgery |
| K733 | Renewal of intravenous biventricular cardiac pacemaker | Pacemaker surgery |
| K732 | Renewal of intravenous dual chamber cardiac pacemaker system | Pacemaker surgery |
| K731 | Renewal of intravenous single chamber cardiac pacemaker system | Pacemaker surgery |
| K741 | Renewal of single chamber cardiac pacemaker system NEC | Pacemaker surgery |
| K722 | Resiting of lead of subcutaneous cardioverter defibrillator | Pacemaker surgery |
| K748 | Cardiac pacemaker system | Pacemaker surgery |
| K749 | Cardiac pacemaker system | Pacemaker surgery |
| K597 | Cardioverter defibrillator introduced through vein | Pacemaker surgery |
| K723 | Subcutaneous cardioverter defibrillator | Pacemaker surgery |
| K728 | Subcutaneous cardioverter defibrillator | Pacemaker surgery |
| K721 | Implantation of subcutaneous cardioverter defibrillator | Pacemaker surgery |
| K729 | Unspecified other cardioverter defibrillator | Pacemaker surgery |
| K738 | Other specified other cardiac pacemaker system introduced through vein | Pacemaker surgery |
| K739 | Unspecified other cardiac pacemaker system introduced through vein | Pacemaker surgery |
| L048 | Other specified open operations on pulmonary arterial tree | Major open |
| L041 | Pulmonary thromboendarterectomy | Major open |
| L049 | Unspecified open operations on pulmonary arterial tree | Major open |
| L284 | Endovascular insertion of stent for aortic dissection in any position | Major endovascular |
| L283 | Endovascular insertion of stent for thoracic aortic aneurysm | Major endovascular |
| L274 | Endovascular insertion of stent graft for aortic dissection in any position | Major endovascular |
| L273 | Endovascular insertion of stent graft for thoracic aortic aneurysm | Major endovascular |
| K011 | Allotransplantation of heart and lung | Major open |
| K021 | Allotransplantation of heart NEC | Major open |
| K028 | Other specified other transplantation of heart | Major open |
| K018 | Other specified transplantation of heart and lung | Major open |
| K024 | Piggyback transplantation of heart | Major open |
| K012 | Revision of transplantation of heart and lung | Major open |
| K026 | Revision of transplantation of heart NEC | Major open |
| K029 | Unspecified other transplantation of heart | Major open |
| K019 | Unspecified transplantation of heart and lung | Major open |
| K022 | Xenotransplantation of heart | Major open |
| K261 | Allograft replacement of aortic valve | Major open |
| K251 | Allograft replacement of mitral valve | Major open |
| K281 | Allograft replacement of pulmonary valve | Major open |
| K271 | Allograft replacement of tricuspid valve | Major open |
| K341 | Annuloplasty of mitral valve | Major open |
| K342 | Annuloplasty of tricuspid valve | Major open |
| K343 | Annuloplasty of valve of heart NEC | Major open |
| K265 | Aortic valve repair NEC | Major open |
| K346 | Closure of pulmonary valve | Major open |
| K345 | Closure of tricuspid valve | Major open |
| K344 | Excision of vegetations of valve of heart | Major open |
| K255 | Mitral valve repair NEC | Major open |
| K312 | Open aortic valvotomy | Major open |
| K311 | Open mitral valvotomy | Major open |
| K314 | Open pulmonary valvotomy | Major open |
| K313 | Open tricuspid valvotomy | Major open |
| K382 | Operations on chordae tendineae | Major open |
| K383 | Operations on mitral subvalvar apparatus | Major open |
| K381 | Operations on papillary muscle | Major open |
| K318 | Other specified open incision of valve of heart | Major open |
| K348 | Other specified other open operations on valve of heart | Major open |
| K268 | Other specified plastic repair of aortic valve | Major open |
| K258 | Other specified plastic repair of mitral valve | Major open |
| K288 | Other specified plastic repair of pulmonary valve | Major open |
| K278 | Other specified plastic repair of tricuspid valve | Major open |
| K308 | Other specified revision of plastic repair of valve of heart | Major open |
| K263 | Prosthetic replacement of aortic valve | Major open |
| K253 | Prosthetic replacement of mitral valve | Major open |
| K283 | Prosthetic replacement of pulmonary valve | Major open |
| K273 | Prosthetic replacement of tricuspid valve | Major open |
| K285 | Pulmonary valve repair NEC | Major open |
| K362 | Pulmonary valvectomy | Major open |
| K264 | Replacement of aortic valve NEC | Major open |
| K254 | Replacement of mitral valve NEC | Major open |
| K284 | Replacement of pulmonary valve NEC | Major open |
| K274 | Replacement of tricuspid valve NEC | Major open |
| K275 | Repositioning of tricuspid valve | Major open |
| K302 | Revision of plastic repair of aortic valve | Major open |
| K301 | Revision of plastic repair of mitral valve | Major open |
| K304 | Revision of plastic repair of pulmonary valve | Major open |
| K303 | Revision of plastic repair of tricuspid valve | Major open |
| K276 | Tricuspid valve repair NEC | Major open |
| K361 | Tricuspid valvectomy | Major open |
| K319 | Unspecified open incision of valve of heart | Major open |
| K269 | Unspecified plastic repair of aortic valve | Major open |
| K259 | Unspecified plastic repair of mitral valve | Major open |
| K289 | Unspecified plastic repair of pulmonary valve | Major open |
| K279 | Unspecified plastic repair of tricuspid valve | Major open |
| K309 | Unspecified revision of plastic repair of valve of heart | Major open |
| K262 | Xenograft replacement of aortic valve | Major open |
| K252 | Xenograft replacement of mitral valve | Major open |
| K282 | Xenograft replacement of pulmonary valve | Major open |
| K272 | Xenograft replacement of tricuspid valve | Major open |
| K349 | Other open open heart valve NOS | Major open |
| K118 | Other specified repair of defect of interventricular septum | Major open |
| K114 | Primary repair of defect of interventricular septum NEC | Major open |
| K112 | Repair of defect of interventricular septum using pericardial patch | Major open |
| K111 | Repair of defect of interventricular septum using prosthetic patch | Major open |
| K113 | Repair of defect of interventricular septum using tissue graft NEC | Major open |
| K117 | Repair of interventricular septal defect using intraoperative transluminal prosthesis | Major endovascular |
| K116 | Repair of multiple interventricular septal defects | Major open |
| K115 | Revision of repair of defect of interventricular septum | Major open |
| K119 | Unspecified repair of defect of interventricular septum | Major open |
